# Sustainable career and employability of student leaders in China

**DOI:** 10.3389/fpsyg.2022.1033401

**Published:** 2022-10-31

**Authors:** Bin Bai, Mixue Li, Xinshan Lyu

**Affiliations:** ^1^Faculty of Education, Beijing Normal University, Beijing, China; ^2^School of Education, University of Nottingham, Nottingham, United Kingdom; ^3^Beijing Municipal Education Commission, Beijing, China

**Keywords:** student leaders, employability, social competence, emotional competence, action competence, responsibility and resilience

## Abstract

Research has shown that most employers prefer employing graduates with student leadership experience in colleges and most of the student leaders have sustainable development in their careers in China. Compared with students without leadership experience, student leaders are easier to get promotion and development opportunities in the workplace. To discuss this question, researchers chose employers, student leaders, and teachers as research participants in an effort to answer the question of why employers prefer graduates with experience being student leaders. This study focuses on what key competencies student leaders possess and how their competencies can match the capability requirement of employers. Semi-structured interviews were conducted with 9 outstanding student leaders, 4 class teachers, 2 student affairs administrators, and 8 human resource managers from two vocational colleges in Beijing. We find that student leaders have strong social competence, emotional competence, action competence, responsibility, and resilience which employers value and these competencies are essential to career development in the workplace. These competencies are considered to be helpful to support student leaders to adapt to the real work environment quickly and achieve career success.

## Introduction

How to foster qualified graduates for the labor market and match or meet the employer’s requirements have always been of attention and a challenge in higher education (Braun and Brachem, 2015). Employers expect higher educational institutes to train their students to acquire, not only theoretical and technical knowledge but also competencies to be able to work in the workplace and create values and profits immediately after graduates enter the labor market (Harvey, 2000). It has been argued that higher educational institutes should not merely provide theoretical knowledge and practical knowledge but help students to apply the knowledge to different work environments and know how to make good use of knowledge to deal with different work tasks under different situations (Hernández-March et al., 2009). Whether graduates can do well on the job largely depends on what competencies they have acquired in formal education institutions (Heijke et al., 2003). Competence has become one of the important approaches to measure if graduates are competent in a job and suitable for the job market and to learn how to foster students to fit in the future job market.

Research has shown that student leaders have a positive impact on college student groups and organizations and their leadership and employability are developed through engaging student management work (All-China Students’ Federation, 2006). The experience of having been a student leader has become one of the important elements for employers to select competent future employees, which has provided many priorities in the job market for graduates to get employed and higher salaries (Cui and Wu, 2018). Data shows that 69.84% of employers prefer employing graduates with student leadership experience in college or university (Fan et al., 2011). It represents that there is a fit of the competences between what employers expect in workplace and what student leaders acquire through working experience in education institutions. The research of student leaders can provide us a good example of exploring what competences employers demand and expect from graduates to do a good job in the workplace and how colleges can train students to acquire these competences and experience the successful transition from higher education institutes to workplace. To discuss these competences, the perspectives and views of different stakeholders, such as employers, student counselors, and student leaders should be included in the research (Mulder, 2017; Löfgren et al., 2020). Therefore, we chose employers, student leaders, and teachers who are in charge of student affairs as our research participants in an effort to answer the question of why employers prefer graduates with experience as student leaders.

To answer this research question, we did research about what competences a good student leader possesses through doing different types of managing work and what competences employers expect from graduates in the workplace, thus, exploring how these two areas can match up. In this research, we will start with the concept of competence to have a brief discussion about different understandings of the nature of competence based on different approaches, thus clarifying how we understand competence in this paper. Then we will outline the literature about the competencies of student leaders and employers’ requirements for the competencies of graduates. The qualitative methodology is used to explore the relationship between student leaders’ competencies and employers’ expectations. This research expects to add more comprehensive knowledge in the field of student leadership and the demands in the workplace based on different views from various stakeholders.

## Literature review

### The concept of competence

The concept of competence is understood and defined in various approaches and perspectives (Le Deist and Winterton, 2005). The behaviorist approach considers competence as the observable and predefined work performances and outcomes (Bound and Lin, 2013). This approach was promoted by McClelland (1998) who questioned the traditional approach to intelligence and demonstrated that competence could be acquired through learning and training. Competence in this perspective is work-orientated, related to the superior, successful, and effective behaviors to perform specific work tasks, and can be tested and assessed through observable work behaviors (Spencer and Spencer, 1993). This competence model is widely used in the field of human resources and management (Bharwani and Talib, 2017). On the other side, competence is regarded as personal characteristics and traits to enable individuals to acquire knowledge, and process information, thus producing good performance (Mulder et al., 2007). It is a more worker-oriented approach. These two sides of understanding competence have separated employees and work as two different entities, considering competence as fixed and static work performances or personal capabilities. It is criticized to be over-simplistic, has narrowed down the nature of competence, therefore, ignoring the complexity of work environment and situations (Norris, 1991; Sandberg, 2000; Bound and Lin, 2013). When the work environment and situation change, workers cannot really adapt to the changes and complete the tasks efficiently and flexibly (Nybø, 2004).

In this paper, competence is no longer mere work performance or personal characteristics, they are integrated into each other. When one person works in different circumstances, they always combine their certain personal abilities and certain work tasks into the contexts to work more appropriately and effectively (Sandberg, 2000). Competence includes knowledge, skills, personal traits, and attitudes, but is not limited to it, it represents how individuals interact with and adapt to different, complex, and changing work environments and situations to generate appropriate actions to solve different problems. Competence is embedded in work contexts. It is not fixed and static, instead, it is always evolving and developing.

### The key competencies of student leaders

Student leaders are students who lead or manage formal or informal student groups or organizations in schools, colleges, and universities (Lyu, 2001). Student leaders, the existence of which is a major feature of the Chinese formal education system, are important organizers, coordinators, and implementers of education, management, service, and other student-related issues. Student leaders mainly serve the classes, student clubs, Communist Youth League organizations, student unions, and associations. As a bridge that links students on the one side and teachers and schools on the other side, student leaders play a key role in representing and serving the majority of students and uniting and connecting student groups (All-China Students’ Federation, 2017). Student leaders are generally responsible for the management of student affairs, the organization of the class and school projects, which are considered to be able to distinguish the outstanding student from ordinary students (Ran, 2011). Being a core member of students in colleges can help students to improve their competencies to adapt to future real work environments better and quicker (Seemiller, 2018; Lundin et al., 2021). To explore why employers prefer to hire graduates with the experience of being a student leader, we need to talk about what is required by being a student leader or what competencies graduates can acquire through student leaders’ experience in college.

Most of the existing studies have adopted quantitative research methods and studied the competencies of student leaders through differential testing and analysis of school, grade, gender, years of service, and other factors. In 2009, NACA (National Association for Campus Activities) published the competency guide for being ideal student leaders (Brill et al., 2009). The identified key competencies are leadership development, assessment and evaluation, event management, meaningful interpersonal relationships, collaboration, social responsibility, effective communication, multicultural competency, intellectual growth, and clarified values. Researchers also added some other competencies as an addition for being a good student leader, which is: enhanced self-esteem, realistic self-appraisal, healthy behavior, satisfying lifestyles, interdependence, spiritual awareness, personal and educational goals, and career choices. Besides the development of leadership, student leaders can also develop other important competencies through organizing and managing the activities, for example, social competencies (interpersonal competencies, communication competencies (Lau et al., 2014; Aymoldanovna et al., 2015) where they can develop a good relationship with other students, staff, employers and so on. From the perspective of organizations, student leaders need to be aware of the values and vision of institutions and know how to serve and benefit the general students, thus make sure the healthy development of the institution (Camacho et al., 2021). Researchers also believe that personal traits, for example, managing emotions, self-management, authenticity, and a sense of justice are also important competencies of being good student leaders (Shen and Liu, 2009; Ran, 2011; Zeng, 2014; Camacho et al., 2021).

Previous research has initially outlined the important competencies of what a good student leader needs to be. However, these questionnaires and surveys with large-scale mainly listed the important competencies and give a definition and description of each competence independent from work contexts, which are difficult to illustrate the dynamics and complexity of contexts and work tasks (Teichler, 2007). It is difficult to explain how and why these competencies are acquired. Based on previous research findings, we conducted in-depth interviews with student leaders, student counselors, and student affairs administrators to explore what is required to be a competent student leader.

### Employers’ requirement to graduate students’ competences

No consensus has been found in the literature about what competencies are most important to employers. Typically, questionnaires and surveys are used to ask employers to list and rank the importance of different competencies they think to recruit an employee for the company. We reviewed different literature to analyze the important competencies that graduates need to have in employers’ view. Research has shown that employers have attached much importance on domain-specific competencies, for example, specific professional technical competence, and work-related experience (Sharma, 2009). Knowing technical concepts and applying them to practical work contexts, for example, knowing the principles of using certain tools and technologies, and knowing how to use certain tools effectively and appropriately in different ways, can enable employees to solve the problems efficiently they meet in different work contexts and situations (Autio, 2011). Besides, non-domain-specific competencies or domain-general competencies also got much attention from employers, sometimes even considered more important than domain-specific competence (Cook and Finch, 1994; McMurray et al., 2016). With the rapid change in the market and work environment, especially in the era of industry 4.0, it seems that employers favor employing graduates who can flexibly adapt to the changes, learn and think independently and systematically, and are creative and innovative (Wang and Liu, 2014; Wen and Jiang, 2016). The reason why employers prefer those competencies is that they enable employees to be competent to engage in different work tasks, thus contributing to the profit of the company immediately (Bennett, 2002). Employers expect graduates to have certain social competence as well, for example, competence to work in a team, cooperate, communicate and coordinate at work (Branine, 2008; Yang and Ming, 2008; Liu, 2013; Wen and Jiang, 2016). The competencies of managing time and organizing work tasks to be able to complete each job task well are also listed as important competence in employers’ perspectives (Yang and Ming, 2008). Employers also value personal traits and work-related attitudes, for example, working hard, passion and respect for their job, honesty or responsibility (Liu, 2013; Zhang and Lin, 2015). Positive work attitudes are referred by employers to enable employees to make contributions to the company’s performance and cohesion (Bolino et al., 2012). Research has shown that responsible workers generally have higher motivations to do their work, and thus can work more efficiently in the industry (Ye and Luo, 2009). Besides, work behaviors, for example, serving the company and society, and obeying certain vocational moral values are also emphasized by employers as the important requirement for future employees (Yang and Ming, 2008; He and Liu, 2014).

Previous research has discussed different competencies that being a good student leader requires and the competencies that employers expect in the workplace, however, less research has talked about the links between the competencies required by being student leader and by doing a good job in the workplace. Most of the research applied quantitative research to explore the most important competencies that an excellent student leader and a qualified employee need, however, this approach more or less ignored the complexity of the work environment and competencies. In this paper, we examine the demand for graduates’ competencies from the perspective of the employer and have a systematic exploration of what competencies employers expect graduates to have, thus helping us explain why graduates with the experience of being the student leader in colleges gain the popularity and more opportunities from employers in the job market.

## Research methods

### Data collection

In this research, we used the qualitative approach and conducted interviews with nine outstanding vocational college student leaders, four student counselors, two student affairs administrators from two vocational colleges in Beijing, and eight employers from different companies whose businesses have a great relationship with colleges’ majors setting. Among the nine student leaders we interviewed, three students’ major is refrigeration equipment operation and maintenance, four students’ major is construction technology application and another two students’ major is automatic control of installation and maintenance in buildings. Some of the students had more than one role, four of them had a position in the Communist Youth League, four student leaders were in the student councils, three of them were serving their respective classes, and one was the chairperson of a student table tennis club. All of the six teachers we interviewed had more than five years of experience serving as student counselors or student affairs administrators. Among those employers, there are three project managers, two of them are production managers, three of them are human resource managers and all of them have more than five years of experience in realty management, aerospace electronics, and electronic equipment manufacturing fields. These employers recruit graduates from these vocational colleges every year. They are very familiar with students’ characteristics and they are also in charge of new staff training. Their basic information is listed in Table 1.

**TABLE 1:** Basic information of interviewees and interviews.

Interviewee	Gender	Roles	Duration of interview (min)	Number of characters transcribed
S1	Female	Deputy Secretary of School Communist Youth League Committee	59	11,896
S2	Male	Secretary of Communist Youth League Branch	44	8,800
S3	Female	Secretary of Communist Youth League Branch	52	10,188
S4	Male	President of School Student Council and Class Monitor	53	10,355
S5	Female	Director of Cultural and Sports Department of Faculty Student Council and Director of Communication of Communist Youth League Branch	81	15,441
S6	Male	Director of School Television Station and Class Monitor	57	11,660
S7	Female	Secretary of Communist Youth League Branch	45	11,582
S8	Female	Director of Discipline Inspection Department of School Student Council	45	8,375
S9	Female	Director of Cultural and Sports Department of School Student Council	54	12,571
T1	Male	Student counselor of Grade 1	53	11,345
T2	Female	Student counselor of Grade 2	55	14,342
T3	Male	Student counselor of Grade 2	52	10,280
T4	Female	Student counselor of Grade 3	61	12,423
T5	Female	Teacher of Teaching Affairs Office	51	10,103
T6	Female	Teacher of Teaching Affairs Office	57	12,035
E1	Male	Project manager of realty management company	78	15,172
E2	Male	Human resource manager of aerospace industry company	82	15,568
E3	Female	Production manager of aerospace industry company	69	14,221
E4	Male	Production manager of electronic technology enterprise	57	12,666
E5	Male	Human resource manager of electronic technology enterprise	56	12,420
E6	Male	Human resource manager of electronic technology enterprise	51	11,075
E7	Male	Project manager of construction company	50	11,006
E8	Male	Project manager of realty management company	94	20,405

We started with the questions about key competencies of outstanding student leaders and what employers expect from graduates in the workplace. Interviewees were asked to describe the competencies required of vocational colleges graduates and what employers value most to employ workers. Interviews for this study were conducted from November 2020 to May 2021. It lasted for around 7 months. With the consent of the interviewees, the researchers recorded all the interviews and transcribed all the recordings. The total length of the recording was 22 hand 36 min and the whole transcript has 283,929 Chinese characters.

### Data analysis

We applied qualitative research and used the software Nvivo11.0 as the analyzing tool for data analysis and coding. It involved three stages of coding, namely, open coding, axial coding, and selective coding. Open coding was first used to identify the themes directly from the interview. As the coding process proceeded, more and more similar codes would emerge and these similar codes would be ascribed into the same code, which is the process of axial coding. After, selective coding would be used through the classification of different themes and an analysis framework would be generated (Huberman and Miles, 2002). One example of the coding process is shown in Table 2.

**TABLE 2:** Sample of interview data encoding.

Open coding	Axial coding	Selective Coding
01. Especially love to talk and want to reach out to more people		
02. Act politely and take time to build relations		
03. Frequently reach out and communicate with teachers		
04. Communicate in advance with school functional departments and the lighting and sound company before an event	Communication	Social competence
05. Verbal skills are the most important for organizing Party lectures		
06. Refrain from using intemperate language in conflicts with classmates		
07. Insist on fact-to-face communication to tackle problems		

In determining the axial coding and the selective coding, we mainly have gone through the following few procedures. We first referred to the dimensions commonly mentioned in the Chinese and English literature for preliminary classification of coding. We then analyzed the views of interviewees and determined the encoding category according to the importance of competence and the frequency by which it was mentioned. In the process of analyzing the data, researchers acted as research tools to construct the competencies, meanings, and values together with participants and previous literature.

## Research findings

### Social competence

Social competence is the ability to handle social interactions effectively. It refers to getting along well with others, being able to form and maintain close relationships, and responding in adaptive ways in social settings (Weiner and Craighead, 2010).

A major task for student leaders lies in organizing on/off-campus student events. For certain big events, they need to prepare for a very long term and many aspects are involved in the process. Student leaders need to work with different departments and persons. Their collaboration skills are tested not only in internal coordination scenarios but also in cases of working with other departments and groups. They should be able to demarcate responsibilities clearly, hold members together, and bring solidarity to the team. S5 mentioned her experience of organizing an award ceremony that involved more than one department, *“In the singing competition, I was the host, and there were other team members responsible for the name-list, the queue, and the certificates. We discussed and determined the division of work and the procedure of ceremony.*”

Student leaders should be able to communicate their ideas clearly, reach out to others to achieve consensus, and get the job done through teamwork. They should also have a strong sense of cooperation, readily appreciate the strength of others, assign tasks accordingly and enable high-level collaboration. “*Collaboration is important. Without it, nobody would want to help you. You must cooperate with others, and you cannot finish a particularly difficult task or big campus events on your own.*” (S3)

Data shows that all the employers considered good communication and collaboration to be very important competence in doing work with colleagues, managers, employees, and other company partners. As a technical worker who needs to deal with technology, equipment, or machine, it is also important to be involved in the team and community to share work experience and knowledge with each other, know everyone’s characteristics and expertise, deal with some work conflicts with other people to be able to cooperate with each other well, all of which needs workers to be able to communicate well with other people in the right moment. As one employer in one electronic company said,

*Though you (the worker) are responsible for the technical production, it’s also required by the job that you need to have the communication competence because you need to communicate with your manager, work partners, and someone who works in other departments. From the perspective of future development, you still need to engage yourself into your team because there are hundreds work procedures in the company to produce one piece of work, everyone needs to communicate and cooperate with each other to assure the quality of result, thus, it requires you to have the skills to communicate.*

*——E4, Electronic Company*

In the workplace, because the technical work, for example, the repair of a large machine in the factory, is combined in many different correlated procedures in a different department, the quality of results in each past can affect and be affected by others. It requires cooperation between many different workers to organize work tasks and do the jobs together as a whole.

*Normally, there are two people in one job site to assist and supervise each other. Sometimes, if there are some big projects, all of the workers need to work together. Once, one fire pipe was exploded and the water was leaking through the pipe, there was strong pressure. It would cause enormous loss to company if it could not be stopped immediately. It was important for workers to communicate with each other efficiently and decide that who turns the valve off quickly. It requires good teamwork competence to solve this problem.*

*——E1, Realty Management Company*

Student leaders are responsible for organizing and participating in different activities, which requires student leaders to communicate and cooperate with different people including other students who work in these activities, tutors, employers, and so on. These work tasks are very similar to what employees need to do in the company. Therefore, it’s easier for student leaders to transfer these social competencies to the future work environment, which will help them to be preferred by employers to apply for a job.

### Action competence

Action competent is a personal capacity in which people are committed and passionate about solving a societal issue and has the relevant knowledge and skill about the issue and takes a critical but positive stance toward different ways of solving it (Sass et al., 2020). It is the competence as a human agent to be practiced in complex and authentic social situations (Fontes, 2004).

Student leaders are important organizers of and participants in student activities. They often need to complete tasks assigned by teachers and supervisors in charge of student affairs. During the process, they are able to develop strong organization, coordination, and execution skills.

Execution lays the foundation for the successful completion of a task. Student leaders that are good executors treat tasks assigned by teachers and superiors rigorously and seriously and never muddle along in a careless or perfunctory manner. “*Be credible and reliable*” (S6), “Some student leaders even can finish working tasks *before teacher assign tasks to them. They are very efficient and make no mistake.*” (S8) Excellent student leaders are not only good executors themselves but also lead teams that execute well (Huang et al., 2010). Members are motivated to act swiftly and synergy is generated. Given the arrangements made by teachers or superiors, they can still think critically and independently and propose novel solutions based on the actual conditions and needs of students.

Student leaders sometimes have to deal with emergencies or conflicts and contradictions with their classmates as they try to fulfill their management responsibilities. Student leaders are skilled problem-solvers that maintain good interpersonal relationships with their classmates. If faced with a conflict, S3 said, “*I will try not to use intemperate or over-emotional language. Instead, I will find a third-party mediator, try to communicate calmly, and apologize if necessary to ensure that the event is not affected*.” Students sometimes conflicted with their teachers or peers when two parties have different views or approaches toward a certain matter and both want the other side to accept his/her own way. Outstanding student leaders are good at handling their relationships with classmates. In the process of solving problems, student leaders can act calmly, adapt to circumstances when necessary, and solve conflicts in flexible ways.

Employers require workers to act and deal with problems and challenges in the workplace. Due to the complexity of work, workers cannot always predict all of the problems which happen in work situations. Employers require workers to be able to respond to different problems quickly, and flexibly and deal with new challenges and solve them.

*We have all of the different types of equipment, some of them are old and some of them are quite new. Workers have to find out different strategies to repair different types of machine, rather than dealing with them in the same way. The real work environment is always complex and dynamic, there is no one standard answers to deal with all of the different problems.*

*——E2, Aerospace Company*

*We are in the research and design department, there are lots of different new models of machine which combines lots of different parts and procedures. It requires our workers to find out how to improve the work efficiency by themselves. For example, we needed to work on one product with 360 degree angles, however it was so inefficient to move the product all the time to work through different angles, so we invented a wheel to help us work in different angles, the problem got solved. In the work situations, you probably will face lots of challenges and difficulties, you must have the competence to solve problems with different levels of complexity in different situations. The competence of solving different problems efficiently is one of the most important competences for the company to evaluate a worker when you want to apply higher-level skilled position.*

*——E3, Aerospace Company*

Action competence includes competencies of solving problems and acting quickly to do tasks. Student leaders take charge of every stage of managing activities from planning, assigning tasks to the right people, acting appropriately, and reflecting. All of these require student leaders to have good competence in time management to assure each task in each stage can be done on time. When they organize and do different tasks, they always face many complicated situations with lots of difficulties and challenges that they cannot even expect in advance. It asks student leaders to make appropriate decisions quickly to make sure the success of activities. Through solving these problems, the competence in solving problems effectively and responding to different challenges quickly gets improved. It shows that this competence is highly required in the workplace for workers to finish work tasks. Student leaders have acquired competence through doing real work tasks, which can help them to work effectively and flexibly in a different work environment.

### Emotional competence

Emotional competence is the manifestation of emotionally competent behaviors (Zeidner et al., 2004; Giardini and Frese, 2008; Seal and Andrews-Brown, 2010) that reflect the ability to perceive accurately, appraise, and express emotion and to access feelings when they facilitate thought and understand emotion and emotional knowledge, regulate emotions to promote emotional and intellectual growth (Mayer and Salovey, 1997, p. 10). It includes three dimensions: emotional awareness, emotional understanding, and emotional regulation (Delcourt et al., 2016).

The experience of being student leader can help individuals improve their emotional awareness and better understand others’ emotions. During the process, they are able to develop strong self-control abilities, which will enable them to adjust their mental and emotional state more effectively in the face of difficulties and setbacks.

Student leaders have more opportunities to reach out to their teachers, classmates, and other people than ordinary students. That means they need to deal with more complicated interpersonal relationships. In their work, student leaders often encounter difficulties or conflicts with other students. Young students normally are not stable in mood and may not be able to control their emotions well. These will easily lead to interpersonal conflicts and contradictions sometimes. Outstanding student leaders are able to adjust their emotions and effectively control negative emotions, such as anger and impulsivity. S6 stressed, “*As a student leader, you must control your emotions. Especially when dealing with conflicts, you must not be impulsive. Even if the other person swears at you, you have to bear with it. You cannot directly argue with him/her. If you can’t handle it by yourself, report to the teacher immediately.*”

Outstanding student leaders have empathy and are able to control their own emotions from the perspective of others. When holding meetings with department members or secretaries of the Communist Youth League Branch, S3 “*Facing with challenge from different stakeholders, I can always stay calm and answer their question one by one.*” Emotional control can help student leaders cope with the pressure of work and maintain an objective and calm position to deal with problems.

In the workplace, the competence of controlling one’s own emotions in work to avoid irrational behaviors is very important. Sometimes, employees need to have a stable mood to avoid conflict with customers even if the customers are rude and difficult to communicate with. employees need to act quickly and use peaceful ways to control the mess, solve the problems and meet customers’ demands.

*You are the representative of company, sometimes, if there are some serious problems in your equipment which affect production, customers normally become very angry. They speak crude language and abuse service staff. Some of our new young colleagues cries when they met such situation at first time. What we need to do is to control our emotion, try our best to communicate with customers, satisfy their demands, we cannot quarrel with our customers directly.*

*——E8, Project manager of realty management company*

During organizing and managing student affairs, student leaders are afforded more opportunities to interact with teachers, classmates, and people outside of colleges, for example, employers and workers affiliated with different organizations than general students. Knowing how to tell and feel other people’s emotions and feelings is the basic principle inherent in social interrelationships for student leaders to learn through experience. They always meet similar challenges as what happens in the workplace, for example, conflicts with customers or other people. This requires them to learn how to control their own emotions and deal with conflicts in a stable and rational emotion. Their emotional competence has improved much through dealing with lots of people. This helps them to easily control their own emotions, and analyze and solve problems rationally and efficiently in the workplace.

### Responsibility and resilience

Responsibility is a person or an organization’s consequences of action referring to a moral or legal standard under supervision or judgment of a judging or sanctioning instance in a specific realm or situation (Auhagen and Bierhoff, 2001; Lauermann and Karabenick, 2011). It is a relatively stable personality disposition and a kind of self-judgment with a sense of internal obligation under an internal locus of control (Lenk, 2007). This article mainly focuses on personal responsibility.

As important assistants to teachers, student leaders are often required to participate in student management and event organization and have to undertake various tasks assigned by teachers from time to time. During the process, they have the opportunity to build a sense of responsibility and development initiatives.

Many student leaders mentioned the importance of being responsible and believed that responsibility directly determined a person’s attitude and persistence at work. S7 said: “*Taking the Communist Youth League class for example, before starting, we had lots of preparations to do. No one chose to have dinner first before the preparation job was finished. We did not need others to remind us. I would go to prepare what I need to do directly after my class was over.*” S2 was quite weak physically and often felt sick, but “I *never took a leave of absence because of illness that year, because I felt that I must persist and should not take a leave even if I was sick.*” The sense of responsibility of student leaders comes partly from the trust of their teachers and seniors and partly from the importance they attach to the work itself and the students who participate in the activities. T1, a supervisor, said: “*The words and deeds of our student leaders represent our class as a whole. If there is any problem within the class, I will definitely first get help from student leaders.*” Responsible student leaders are devoted to student managers and fully understand their value in the organization. They have very strong organizational identification and regard themselves as part of the community.

To Being responsible for what employees are doing at work is attached definitely important to employers. Employers prefer to assign more important work tasks to workers with a strong sense of responsibility for their work because they are always able to be responsible to their own actions and behaviors, which is very important for doing well in the work and producing high-quality products. For example, for the work in the aerospace field, it is very important to be responsible for workers what they’ve done, because it is highly related to the safety of people, society, and even the whole environment.

*We need to take much responsibility to work in aerospace field, you must tell yourself anytime to be responsible to your work and products, you cannot play around to just finish the tasks, we are not permitted to make any mistake. Once there are some problems in the future, we need to pay huge price for the irresponsibility. We have to remind ourselves in every moment that we need to be responsible to the quality of our products.*

*——E2, Aerospace company*

Resilience is a relatively stable personality trait characterized by the ability to overcome, steer through and bounce back from adversity (Ong et al., 2006). This ability enables a person to quickly regain equilibrium physiologically, and psychologically in social relations following a stressful event. Assets and resources within the individual, their life and environment facilitate this capacity for adaptation and ‘bouncing back’ in the face of adversity (Windle, 2011).

Student leaders often take on organizing and managing tasks in college. Despite facing the challenging situation, S6 emphasizes, “*Certainly I have to spend much time to train new club members and organize school events, I always meet challenges time by time, but I never give up in adversity. Sometimes your efforts would win the trust of others and you could do a good job together. Though there is no material rewards from the school, I don’t care about it at all. Even some commending words in recognition of your efforts can make me happy, you will think it is worth doing this job.*”

S6 recalled his feelings when being asked to take photos for the school art festival for the first time, “*I knew nothing about photography and it was a complete disaster. I took about two hundred to three hundred photos and most of them were not qualified. At that time, I felt frustrated and my heart was broken. I am not the people who will give up so easily. Later I tried to adjust myself and went to 798 Art District many times to learn to take pictures and finally I become an excellent photographer.*”

To meet the demands of customers and pursue more profits, products need to be produced and sent out to customers in a limited time and the quality of products also needs to be assured. There probably will be lots of difficulties happening in working on different tasks, which requires workers to solve the technical difficulties in the process of production by using limited resources within a short time. Working in this environment has brought much pressure on workers which need workers to solve problems in a short time.

*We are not allowed to make even smallest mistakes in the process of producing every piece, each product must be qualified and standardized. If there is the possibility of 0.0001% that one small part failed to work, the mission will be 100% failed. Our work tasks don’t allow any chance of mistakes, so we are used to finish our work under much pressure.*

*——E4, Electronic Company*

*You have to get the competence to deal with the huge pressure from your work. When you start to become the designer, you cannot ask your mentor anytime, you have to deal with all of the problems independently. Sometime, the customer just calls me and asks me, ‘what happened in the process, why it’s not what I expected from you?’ My hands and whole body just become icy immediately when my customers are not satisfied with my design and products. I must face lots of pressure from different customers with different demands, it is quite normal in work.*

*——E7, Construction Company*

In the process of participating in and organizing different activities, student leaders have established a co-dependency relationship with others. They have realized their responsibility for their work and other people through socialization practice, gradually internalized social values and social values into themselves, and then produce the corresponding responsible behaviors. Compared with general students, they have clearly recognized their own social roles and obligations in organizing these activities through interacting with others. This sense of responsibility can be transferred to work, enabling them to engage in work more proactively and responsibly.

Resilience, as an individual’s ability to cope with stress and actively deal with negative emotions, is correlated with individual subjective well-being and life satisfaction and negatively related to depression, anxiety, and other negative emotions. In the process of organizing student activities, student leaders often face much pressure. They are more inclined to actively seek organizational and social support, thus cultivating strong psychological resilience. The transfer of this ability to work will help them actively seek support from others, listen and accept suggestions carefully, complete work tasks better, and thus achieve higher work efficiency. In the face of pressure at work, the ability can help student leaders to reasonably understand and interpret the work content to cope with changes in the dynamic environment, so as to construct a more protective environmental system, thereby promoting their development in the workplace.

## Conclusion and discussion

### Competence matching model from student leaders to employees

**FIGURE 1:**
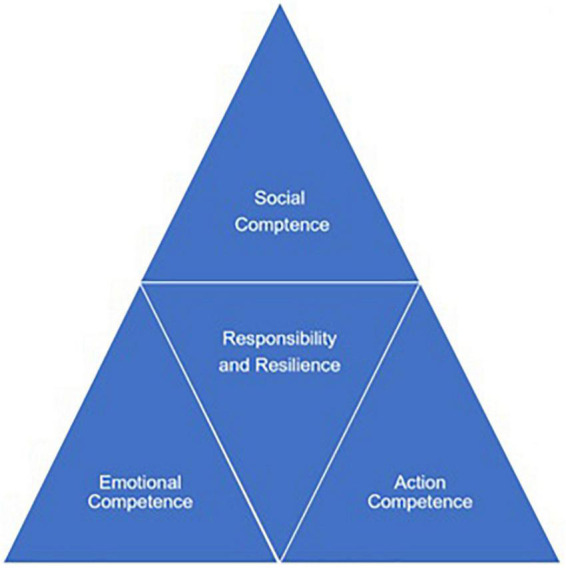
Competence matching model from student leaders to employees.

Researchers have designed a model for competence matching from student leaders to employees. In this model (Figure 1), responsibility and resilience are in the middle and they are the most important personality traits which are valued by employers. Social competence, emotional competence, and action competence are very helpful in communicating, collaborating, and developing good personal relationships in the workplace. Action competence help employees solve problems in the complex and authentic work situation.

In this study, we have shown that student leaders have strong social competence, emotional competence, action competence and responsibility, and resilience which employers value and are considered important to do well in the real work environment. These competencies are considered to be helpful to support student leaders to adapt to the real work environment quickly and successfully transit to the workplace from college. We not only build up the matching model and the relationship between being a student leader and an employee, but we also provide an explanation of why they can be correlated to each other through the comprehensive competence approach.

The relationship between the acquisition of key competencies employers prefer and the experiences of student leadership is reflected in three aspects:

The first dimension is that student leaders’ work tasks in vocational colleges are similar to those in employees’ work contexts. Student leaders have been trained through doing management job and engaging themselves in different activities inside and outside the campus. Management job requires students to know and do all-around tasks, not only limited to one single task. Those campus work tasks can not only shape student leaders’ social interpersonal communication but allow students to learn how to deal with different unexpected situations through which their adaptability to the changes in the work environment gets improved. They also get a chance to interact with different people who are not only limited to students and staff in the colleges but also people in different positions from different agencies, institutions, and organizations outside of the college. They can acquire social communication and incorporation competence and know how to apply different strategies to talk to different people. Being a student leader requires students to make decisions and act immediately, doing this job can train them to acquire action competence. As student leaders, they take charge of the whole work process in campus activities. It requires them to be responsible for other people and the things they take care of. Sometimes, they can fail tasks when they are not familiar with, through which they become more resilient to difficulties and failures. Those competencies which are fostered in campus activities can easily transfer to work situations and help student leaders adapt and be competent in their job.

The second dimension is the adaptation of work roles. Student leaders play multiple roles, they are managers and organizers. Compared with other students, they are expected to take on more responsibilities and duties. They are generally assigned tasks with challenges and student leaders must burden their responsibility and duties. College teachers consider student leaders as their good assistants, they place a higher value on what student leaders do and what they can contribute to student affairs. Other students expect that student leaders can be responsible, helpful, supportive, and fair. When some problemshave been found, student leaders are expected to manage their emotions, solve problems and make immediate decisions. Employers and people from other organizations think student leaders should be able to communicate with them and organize things well. All of the stakeholders have afforded many responsibilities and expectations to the role of student leaders. Taking the role of student leaders means that their behaviors are regulated in certain ways. Being a student leader can help students to recognize their specific social and work roles and be aware of the role behaviors under this role. Through this process, student leaders need to adjust their role awareness and role behaviors all the time. Therefore, this student leadership experience can train these students to quickly define their roles, build specific role awareness and adjust their work behaviors to what employers expect from them when they enter the work environment. Employers have good reasons to favor such kinds of employees and they are easy to get promotions in career development.

The third dimension is that student leaders intend to develop their work values and career identity through student leadership work experience. As it’s mentioned in our findings, student leaders have developed strong beliefs and accredited much value to what they do. Student leaders find and recognize the value embedded in the work itself rather than external rewards. They have built up their work identities through working, for example, interacting with students, solving problems, learning new techniques, and so on. We can say that student leaders are willing to devote themselves to the work of student administration and serving students voluntarily. This strong belief and responsibility for the work itself are highly valued by employers who always try to build up the culture and environment to cultivate employees’ work identities. The work identity can support workers to work more efficiently and actively and encourage them to strive for better achievements in their work.

### Colleges should foster all students’ employability and leadership

Every student has to face the challenges of entering the labor market when they graduate from college. Students’ employability is the key to finding suitable work and colleges should foster every student’s social competence, emotional competence, action competence, and responsibility and resilience during their stay in the colleges.

It’s difficult to equip students with these competencies through delivering different courses and teaching students, for example, how to communicate and cooperate with different people by telling them different personalities and providing different tips. Educational institutions need to change the theory-based methods and strategies, rather than apply the method of ‘learning by doing, by experiencing, facilitating student’s learning, and improving student’s employability through real engagement and experience (Laukkanen, 2000). It’s suggested that universities/colleges involve these competencies in the curriculum design, for example, organizing students to do projects in teams to help students learn how to communicate and cooperate with others, asking students to engage in the process of managing, organizing, designing a project to train students to be able to manage different projects and improve their leadership, set time limits to improve students’ ability of time management and so on. It also supports of training students to acquire these competencies by motivating students to engage in extracurricular activities outside the classroom where most student leaders acquire these competencies. Student leaders are able to see the whole picture of different projects and work on different parts to know the knowledge, colleges should provide opportunities to each student to be involved in a different part of work to know the whole knowledge through doing different work tasks. The designed work tasks should be modeled in accordance with real work tasks in the real work environment to cross the boundaries between colleges and real workplaces which can help students successfully complete their transition from college to the real work environment (Arranz et al., 2017).

### Student leaders should strive to develop professional competence

The experience of assisting in the management of student affairs, although of great help to the development of social and interpersonal skills and other key competencies, contributes little to the academic performance of student leaders as it is hardly related to professional training. Employers want those who can bring value to the organization and attach greater importance to professional competencies and solutions (Sharma, 2009). Student leaders need to spend more time, time that may have been spent on study, at work than ordinary students. If they fail to plan properly, their academic performance will be affected. Some student leaders spend too much time and energy at work and fail exams. Therefore, it is necessary to warn student leaders about the danger of fulfilling positional duties at the expense of a major’s learning. They must take knowledge and professional training seriously, as professional competencies form the foundation of their future career path.

Student leaders usually enjoy broader horizons than ordinary students because their experience in managing student affairs enables them to meet more people and handle more complex situations. However, some students may identify themselves as privileged “student officers”. Their bureaucratic way has been widely criticized. Some students become arrogant and complacent when promoted from junior executive positions to senior managerial positions. These student leaders are not welcome by the job market and they need to adopt the right attitude toward equality and a calm mind in handling their work and relationships with others. They should learn to see from the perspective of others and avoid falling into the abyss of power. Some student leaders continue to have an inflated view of their own importance after leaving school and starting their careers. They believe that simple tasks and daily routines are beneath their notice but lack the qualities that managers expect of good employees and that are critical to career success such as diligence, a down-to-earth attitude, and a steady mind. Therefore, it is also necessary to warn student leaders that a down-to-earth work attitude is always valuable no matter how senior their positions have been in the student organizations and what remarkable achievements they have made. When they join a company, they need to keep their feet on the ground, start from the basics, abide by the rules, regulations, and management instructions of the organization, and fulfill their job duties in a down-to-earth manner.

### Limitations of this study and future research

In this research, we have adopted qualitative research methodology to explore the competencies that employer require in the job market and what student leaders have acquired through their management tasks and we try to provide an explanation of why students with leadership experience are preferred in the job market. We have constructed a competence model in this research; however, the number of samples may limit the generalization of this model. This research mainly is conducted in Chinese contexts, it also may influence the generalization of this model and the application of this model to other socio-cultural and educational contexts in other countries. We suggest employing more samples in the future and using quantitative research methodology to test this model’s effectiveness. Future research should also consider the broad international contexts in this field.

We mainly employed student leaders from vocational colleges and explored their experience and how they acquired competencies through doing leadership tasks. In terms of students’ specific characteristics, there may be some differences existed between student leaders in vocational colleges and those in universities, so our conclusions might limit the application of the model to universities. Future research is suggested to consider the difference in students’ characteristics, curriculums, and cultures in vocational colleges and universities.

In this research, we constructed a competence-matching model in this research which includes four aspects: responsibility and resilience, social competence, emotional competence, and action competence. In the future, how each competence is related to employability and student leadership can be explored in more detail.

## Data availability statement

The original contributions presented in this study are included in the article/supplementary material, further inquiries can be directed to the corresponding authors.

## Ethics statement

Ethical review and approval was not required for the study on human participants in accordance with the local legislation and institutional requirements. The patients/participants provided their written informed consent to participate in this study.

## Author contributions

BB participated in conceptualization, supervision, the design of experimental methods, analysis of experimental data, visualization of experimental results, and the revising of the manuscript. ML participated in the supervision and was also responsible for the specific communication, manuscript revision, submission, and publication. XL participated in the actual investigation, experimental data analysis, experimental results visualization, and the writing of the first draft of the article. All authors contributed to the article and approved the submitted version.
